# Association between socioeconomic status and obesity in a Chinese adult population

**DOI:** 10.1186/1471-2458-13-355

**Published:** 2013-04-17

**Authors:** Yuanyuan Xiao, Naiqing Zhao, Hao Wang, Jie Zhang, Qingfang He, Danting Su, Ming Zhao, Lixin Wang, Xinwei Zhang, Ruying Hu, Min Yu, Zhen Ye

**Affiliations:** 1 Zhejiang Provincial Center for Disease Control and Prevention, Hangzhou, Zhejiang, China; 2 Zhejiang Health Bureau, 216 Qingchun Road, Hangzhou, Zhejiang, China; 3 School of Public health, Fudan University, Shanghai, China

**Keywords:** Socioeconomic status, Obesity, Association, Cross-sectional study

## Abstract

**Background:**

Existing studies which regarding to the association between individual socioeconomic status (SES) and obesity are still scarce in developing countries. The major aim of this study is to estimate such association in an adult population which was drawn from an economically prosperous province of China.

**Methods:**

Study population was determined by multilevel randomized sampling. Education and income were chosen as indicators of individual SES, general obesity and abdominal obesity were measured by body mass index (BMI) and waist circumference (WC). Descriptive statistical methods were used to depict overall and factor-specific distributions of general and abdominal obesity among 16,013 respondents. Two-step logistic regression models were fitted on gender basis.

**Results:**

The age-and-sex adjusted rates of general overweight, general obesity, abdominal overweight and abdominal obesity in study population were 28.9% (95%CI: 27.9%-29.9%), 7.5% (95%CI: 7.0%-8.1%), 32.2% (95%CI: 31.2%-33.3%) and 12.3% (95%CI: 11.6%-13.1%), respectively. Based on model fitting results, a significant inverse association between education and obesity only existed in women, while in men, income rather than education was positively related to obesity.

**Conclusions:**

The atypical SES-obesity relationship we found reflected the on-going social economy transformation in affluent regions of China. High-income men and poorly-educated women were at higher risk of obesity in Zhejiang province, thus merit intense focuses.

## Background

Obesity has rapidly developed into a global health challenge during last two decades. According to the World Health Organization (WHO), between 1980 and 2008, the worldwide prevalence of obesity (defined by body mass index, BMI ≥ 30 kg/m^2^) almost doubled. By the end of the year 2008, 1.5 billion adults had passed the threshold of overweight, more than one third of them were actually obese [1], and in every five obese individuals, there was a Chinese [2]. It has been well proved that obesity can be a major risk factor to various diseases, especially for coronary heart disease and ischemic stroke [3-5]. Every year, at least 2.8 million adults die as a result of being overweight or obese [1]. Previous studies have disclosed that among obese individuals, even moderate weight loss could bring significant benefits to health [6,7]. Thus, the identification of obesity risk factors is vital to the development of intervention policies and measures.

Before 1990s, the etiologic studies of obesity mainly focused on biological explanations, very few of them dedicated to explore social and psychological causes. Ever since Sobal and Stunkard’s far-reaching review [8], the relationship between socioeconomic status (SES) and obesity had quickly become the hot spot of research. Most studies addressing this issue were implemented in developed countries, and the majority of them confirmed the pattern which was concluded by Sobal and Stankard, that is an inverse association between individual SES and obesity was often observed in women. But in developing countries, the total number of SES-obesity study remains minimal, not even mention about the fact that the results varied considerably between studies [9-11].

The major aim of our study is to estimate the independent associations between different individual SES indicators and obesity in study population. In order to better achieve this goal, when constructing statistical models, we controlled for various possible confounders.

## Methods

### Study population

*Zhejiang metabolic syndrome prevalence survey* was conducted in the year 2010. Multistage randomized sampling method was used to choose participants. Before sampling, we used cluster analysis to categorize all 90 counties in Zhejiang province (which resides in the eastern coastal area of China) into 5 groups based on their regional economy performances. There were altogether 4 stages in our sampling process. At first, we randomly chose out 3 counties in each of these 5 groups, after that, we sampled 4 streets or townships in each of the chosen counties. Then, 3 communities or villages were determined in each of the chosen streets or townships. In the last stage, cluster sampling method was used to collectively draw 40 households in each of the chosen communities or villages. Family members of the selected households whose age were no less than 18 years at the survey day were eligible subjects. Totally 19,113 individuals were selected (required sample size is 15,369), 1,676 of them failed to complete the study (either because of unwilling to attend from the beginning, or dropped out in the midway, or could not be reached all along), finally, we have successfully recruited 17,437 subjects, and the response rate was 91.2%.

Study protocol was approved by institutional review board of Zhejiang Provincial Center for Disease Control and Prevention.

### Measurements

Indoor face to face interview was applied to collect detailed information from all respondents by using a self-developed questionnaire. The questionnaire consisted of two parts: brief profile of the whole family, like household income, and detailed information of respondents, like demographic characteristics (age, gender, ethnicity, education level, marital status, etc.) and lifestyle factors (smoking, alcohol intake, physical activity status and dietary habits, etc.). Before interview, all respondents provided informed written consents.

Anthropometric indexes such as height, body weight and waist circumference (WC) were measured by intensely trained field workers the day after indoor interview. Height and weight were collectively measured by an integrated measuring scale (manufacturer: Wuxi weigher factory Co., LTD., type: ZT-120) , height was measured to the nearest 0.1 centimeter (cm), body weight was measured to the nearest 0.1 kilogram (kg), all subjects were required to remove shoes, coats and other belongings before measurement. WC was measured to the nearest 0.1 cm at the midpoint between the lower rib margin and the iliac crest by a plastic scale tape (manufacturer: Guangdong Wintape Co., LTD., type: not specified). In order to reduce measuring error, WC was measured three times to calculate the mean value for each subject.

### Variables and definitions

Perceived that WHO obesity cut-offs might not be applicable to Asians [12,13], in the year 2003, the Working Group on Obesity in China (WGOC) forwarded exclusive obesity cut-offs for Chinese people [14]. In this study, we used WGOC’s criteria to measure general obesity and abdominal obesity. BMI was calculated as weight (kg) divided by height squared (square meter, m^2^), general overweight was defined as 24.0 kg/m^2^ ≤ BMI < 28.0 kg/m^2^, general obesity was defined as BMI ≥ 28.0 kg/m^2^. Abdominal overweight and obesity were gender-specific: 80 cm ≤ WC < 90 cm and WC ≥ 90 cm for women, 85 cm ≤ WC < 95 cm and WC ≥ 95 cm for men. For extended comparison, we also calculated general overweight and obesity rates based on WHO cut-offs: 25.0 kg/m^2^ ≤ BMI < 30 kg/m^2^ for defining general overweight, and BMI ≥30 kg/m^2^ for defining general obesity [15].

Individual SES was measured separately by education level and per capita household yearly income. Education level was defined as: primary or lower (≤6 years), secondary (7 to 12 years) and advanced (≥13 years). Per capita household yearly income was divided into three subgroups: high (more than 20,000 RMB, 1 RMB is approximate to 0.15 dollar under current exchange rate), medium (10,000 to 19,999 RMB) and low (below 10,000 RMB).

Detailed information on dietary habits was collected simultaneously, which included dining portion and frequency of common ingredients. Among all these ingredients, we mainly focused on three categories in analyses: total fat, total meat, fruits and vegetables. The main reason to pay special interest in fruits and vegetables intake is that, in this study, daily energy intake information was unavailable, and a previous study has found that fruits and vegetables intake can be a plausible surrogate for daily energy intake among Chinese [16]. As to total fat and total meat intake, it has been reported that both of them were closely related to obesity [17,18]. To each of these three categories, we dichotomized all respondents into “high intake” and “low intake” subgroups by the median of average daily intake amount. As to physical activity level, subjects who were engaging in intense physical activities related professions (such as manual worker, farmer or athlete) were defined as “active” ones, regardless of the amount of intentional physical activities in their spare times. To the rest respondents, based on the criteria which were forwarded by Commonwealth Department of Health and Aged Care of Australia [19], who either did no less than 150 minutes moderate physical exercises per week or no less than 60 minutes intense physical exercises per week was classified into “active” group, otherwise was classified into “inactive” group. Marital status included: single, married, divorced and widowed. Smoking or alcohol intake was labeled as: non-smoker/drinker, ex-smoker/drinker and present-smoker/ drinker.

### Statistical analysis

Descriptive analysis was used to illustrate distributive features of study population. Continuous variables were presented as “mean (standard deviation, s.d.)”, categorical variables were given as “point estimate (95% confidence interval, CI)”. Inter-sex and inter-subgroup differences were examined by *t* test or Chi-square test as appropriate to variables.

Multivariable logistic regression model was applied to estimate associations between SES indicators and obesity. Considering gender may have interactions with many other variables, models were fitted by men and women separately. For each gender, we fitted four models, in model 1 and model 2, we took education level as SES indicator, to estimate its association with each of the following two dependents: general overweight and obesity (defined as BMI ≥ 24 kg/m^2^), abdominal overweight and obesity (defined as WC ≥ 85 cm for men and WC ≥ 80 cm for women). While in model 3 and model 4, SES indicator was replaced by income level. Considering that lifestyle factors may lie in the causal pathway between SES and obesity, we introduced a two-step strategy in independents choosing. In step one, besides corresponding SES indicator, we only put age, age^2^ and marital status into the model, and in step two, we added in other lifestyle factors, which included: smoking, alcohol intake, physical activity level, total fat intake, total meat intake, fruits and vegetables intake.

All reported *p* values were based on two-sided test, and the significance level was placed at 0.05. Logistic regression models were performed by SAS (Version 8e for windows; SAS Institute, Inc. Cary, North CA, USA), all other statistical processes were run by STATA (Version 7.0; Stata cooperation; College Station, Texas, USA).

## Results

### Characteristics of study population

After the exclusion of incomplete and ambiguously categorized observations, finally we got 16,013 subjects to analyze. The general features of deleted observations were comparable to remnants (data not shown). Distributive features indicated that older adults took the majority of study population, with an age mean of 49.4 years (s.d. = 15.1 years). The grand mean of BMI was 23.2 kg/m^2^ (s.d. = 3.3 kg/m^2^). The overall crude rates of general overweight and obesity were 29.9% (95%CI: 29.2%-30.6%) and 7.6% (95%CI: 7.2%-8.0%) by WGOC cut-offs, compared with 24.1% (95%CI: 23.4%-24.8%) and 2.9% (95%CI: 2.6%-3.1%) by WHO cut-offs. The crude rates of abdominal overweight and obesity were 31.3% (95%CI: 30.6%-32.0%) and 11.9% (95%CI: 11.4%-12.4%), with a significant inter-gender difference in abdominal obesity: 13.9% (95%CI: 13.2%-14.6%) in women compared with 9.7% (95%CI: 9.0%-10.4%) in men (χ^2^ = 67.02, *p* < 0.001) (Table 1).

**Table 1:** **Characteristics of study population, Zhejiang metabolic syndrome prevalence survey, China, 2010 **^**a**^

**Characteristics**	**Both genders (N = 16,013)**	**Men (N = 7,422)**	**Women (N = 8,591)**	***p *value **^**b**^
Age (year)	49.4(15.1)	50.3(15.3)	48.6(15.0)	<0.01
Weight (kg)	60.0(10.5)	64.3(10.3)	56.2(9.1)	<0.01
Height (cm)	160.7(8.0)	166.2(6.6)	156.0(5.9)	<0.01
Body mass index (kg/m^2^)	23.2(3.3)	23.2(3.2)	23.1(3.4)	<0.01
General overweight ^c ^(%)	24.1(23.4-24.8)	24.9(24.2-25.6)	23.5(22.8-24.2)	0.04
General overweight ^d ^(%)	29.9(29.2-30.6)	31.3(30.6-32.0)	28.7(28.0-29.4)	<0.01
General obesity ^c ^(%)	2.9(2.6-3.1)	2.5(2.1-2.9)	3.2(2.8-3.6)	<0.01
General obesity ^d ^(%)	7.6(7.2-8.0)	7.4(7.0-7.8)	7.9(7.5-8.1)	0.23
Abdominal overweight (%)	31.3(30.6-32.0)	31.0(29.9-32.1)	31.5(30.5-32.5)	0.48
Abdominal obesity (%)	11.9(11.4-12.4)	9.7(9.0-10.4)	13.9(13.2-14.6)	<0.01
Education level (%)				<0.01
Primary or lower	52.0(51.2-52.7)	46.3(45.1-47.4)	56.9(55.8-58.0)	
Secondary	43.3(42.5-44.1)	48.6(47.5-49.8)	38.7(37.7-39.7)	
Advanced	4.7(4.4-5.1)	5.1(4.6-5.6)	4.4(4.0-4.9)	
Marital status (%)				<0.01
Single	6.8(6.4-7.2)	8.0(7.4-8.6)	5.8(5.3-6.3)	
Married	87.4(86.8-87.9)	88.5(87.7-89.2)	86.4(85.7-87.1)	
Divorced	0.6(0.5-0.7)	0.7(0.5-0.9)	0.4(0.3-0.6)	
Widowed	5.3(5.0-5.7)	2.8(2.5-3.2)	7.4(6.9-8.0)	
Individual SES (%)				0.34
Low	33.2(32.5-34.0)	33.4(32.3-34.4)	33.1(32.1-34.1)	
Medium	28.2(27.5-28.9)	27.7(26.6-28.7)	28.7(27.7-29.7)	
High	38.6(37.8-39.3)	39.0(37.8-40.1)	38.2(37.2-39.2)	
Smoking (%)				<0.01
Non-smoker	69.9(69.2-70.6)	39.2(38.1-40.3)	96.5(96.1-96.9)	
Ex-smoker	7.0(6.6-7.4)	12.0(11.3-12.8)	2.7(2.3-3.0)	
Present-smoker	23.1(22.4-23.7)	48.8(47.6-49.9)	0.9(0.7-1.1)	
Alcohol intake (%)				<0.01
Non-drinker	70.3(69.6-71.0)	46.4(45.3-47.6)	90.9(90.2-91.5)	
Once-drinker	4.7(4.4-5.0)	7.2(6.6-7.8)	2.5(2.2-2.9)	
Present-drinker	25.0(24.5-25.7)	46.4(45.2-47.5)	6.6(6.1-7.2)	
Physical activity status (%)				<0.01
Active	19.3(18.7-20.0)	26.8(25.8-27.8)	12.9(12.2-13.6)	
Inactive	80.7(80.0-81.3)	73.2(72.2-74.2)	87.1(86.4-87.8)	

^a ^Values presented are means or crude rates (with standard deviation or 95% confidence interval).

^b^*p*-values for the differences between genders were based on *t *test or Chi-square test as appropriate.

^c ^Measured by WHO cut-offs.

^d ^Measured by WGOC cut-offs.

Compared with Zhejiang provincial statistics of 2010 [20], our sample showed slight discrepancies in age and sex distributions, so we used direct standardization method to further estimate adjusted rates. The age-and-sex adjusted rates of general overweight and obesity were 28.9% (95%CI: 27.9%-29.9%) and 7.5% (95%CI: 7.0%-8.1%) by WGOC cut-offs, 23.5% (95%CI: 21.9%-25.1%) and 2.8% (95%CI: 2.3%-3.4%) by WHO cut-offs. Adjusted abdominal overweight and obesity rates were 32.2% (95%CI: 31.2%-33.3%) and 12.3% (95%CI: 11.6%-13.1%).

### Distributive features of obesity

Several similarities were observed in the factor-specific distributions of obesity between men and women. In both genders, general overweight and obesity (defined as: BMI ≥ 24 kg/m^2^) was more prevalent among middle aged (45–59 years) individuals, married respondents had a higher proportion of either type of obesity, and high fruits and vegetables intake group had a significantly higher proportion of general overweight and obesity.

However, inter-gender discordances were dominant. For example, in female respondents, abdominal overweight and obesity (defined as: WC ≥ 80 cm) was more popular among older individuals (60 years and above), whereas in men, abdominal overweight and obesity (defined as: WC ≥ 85 cm) was more seen in middle aged subgroup. As to SES-obesity distributions, we found that along with the progress of education level, obesity proportions were drastically decreasing only in women, and by contrast, a positive trend was found between income and obesity proportions only in men. Besides, being physically active and smoking were all inversely related to both types of obesity only in men (Table 2).

**Table 2:** Obesity distributive features by related factors, Zhejiang metabolic syndrome prevalence survey, China, 2010

**Factors**	**Men**		**Women**	
	**General overweight & obesity **^**a **^**N(%)**	**Abdominal overweight & obesity **^**b **^**N(%)**	**General overweight & obesity **^**a **^**N(%)**	**Abdominal overweight & obesity **^**c **^**N(%)**
Age				
18-44 years	981(37.6) ^*^	988(37.9)^*^	845(25.2)^*^	975(29.1) ^*^
45-59 years	1120(42.6)	1149(43.7)	1433(45.2)	1730(54.5)
60 years and above	768(35.1)	882(40.4)	862(41.6)	1195(57.7)
Education level				
Primary or lower	1239(36.1)^*^	1355(39.5)^*^	2080(42.5)^*^	2603(53.2)^*^
Secondary	1492(41.3)	1535(42.5)	995(29.9)	1218(36.7)
Advanced	138(36.5)	129(34.1)	65(17.2)	79(20.8)
Income				
Low	834(33.7)^*^	892(36.0)^*^	1080(38.0)	1313(46.1)
Medium	808(39.4)	818(39.8)	879(35.7)	1074(43.6)
High	1227(42.4)	1309(45.3)	1181(36.0)	1513(46.1)
Marital status				
Married	2644(40.3)^*^	2789(42.5)^*^	2807(37.8)^*^	3459(46.6)^*^
Others ^d^	225(26.3)	230(26.9)	333(28.5)	441(37.7)
Physical activity level				
Active	673(33.8)^*^	703(35.3)^*^	437(39.5) ^*^	503(45.5)
Inactive	2196(40.0)	2316(42.6)	2703(36.1)	3397(45.4)
Total fat intake				
Low	1432(38.7)	1513(40.9)	1554(36.4)	1961(46.0)
High	1437(38.6)	1506(40.4)	1586(36.7)	1939(44.8)
Total meat intake				
Low	1318(36.4)^*^	1410(38.9)^*^	1772(37.4)	2211(46.6)^*^
High	1551(40.8)	1609(42.3)	1368(35.6)	1689(43.9)
Fruits and vegetables intake				
Low	1329(36.1)^*^	1404(38.1)^*^	1432(34.8)^*^	1824(44.4)
High	1540(41.2)	1615(43.2)	1708(38.1)	2076(46.3)
Smoking status				
Present smoker	1277(35.3)^*^	1400(38.7) ^*^	25(34.3)	35(48.0)
Others ^e^	1592(41.9)	1619(42.6)	3115(36.6)	3865(45.4)
Alcohol intake status				
Present drinker	1357(39.5)	1423(41.4)	243(42.8)^*^	310(54.6) ^*^
Others ^f^	1512(38.0)	1596(40.1)	2897(36.1)	3590(44.8)

^a ^Defined as: BMI ≥ 24 kg/m^2^^b ^Defined as: WC ≥ 85 cm ^c ^Defined as: WC ≥ 80 cm.

^d ^Include: unmarried, divorced and widowed ^e ^Include: ex-smoker and non-smoker.

^f ^Include: ex-drinker and non-drinker.

^*^p < 0.05 based on Chi-square test.

### Model fitting results

Among male respondents, a slight decrease was found in adjusted odds ratios of SES indicators after including lifestyle factors into the initial regression models. Income level was significantly associated with obesity after adjustment. Compared with the lowest income group, the adjusted odds ratios of “general overweight and obesity” and “abdominal overweight and obesity” among men with medium level income were 1.20 (95%CI: 1.06-1.35) and 1.15 (95%CI: 1.01-1.29), while among those with high level income, the adjusted odds ratios were 1.35 (95%CI: 1.20-1.51) and 1.42 (95%CI: 1.26-1.59), respectively. Among married men, the odds of general or abdominal obesity were about 1.4 times to those out of marriage. And among physically active men, the odds of general or abdominal obesity were about one quarter less than physically inactive ones. Fruits and vegetables intake amount was positively related to both types of obesity (Table 3).

**Table 3:** **Logistic regression results of men, Zhejiang metabolic syndrome prevalence survey, China, 2010 **^**a**^

**Independents **^**d**^	**General overweight & obesity **^**b**^		**Abdominal overweight & obesity **^**c**^	
SES: Education level^[Contrast: primary or lower]^	Model 1(Step1)	Model 1(Step 2)	Model 2(Step1)	Model 2(Step 2)
Secondary	1.30(1.16,1.45)^**^	1.24(1.11,1.38) ^**^	1.28(1.15,1.42) ^**^	1.21(1.09,1.35) ^**^
Advanced	1.46(1.14,1.87) ^**^	1.27(0.99,1.63)	1.26(0.99,1.62)	1.12(0.87,1.44)
Married ^[Contrast: unmarried, divorced and widowed]^	1.41(1.17,1.70) ^**^	1.40(1.16,1.70) ^**^	1.45(1.21,1.75) ^**^	1.43(1.19,1.72) ^**^
Present smoker^[Contrast: ex-smoker& non-smoker]^		0.66(0.60,0.73) ^**^		0.77(0.70,0.85) ^**^
Present drinker^[Contrast: ex-drinker& non-drinker]^		1.05(0.95,1.16)		1.02(0.93,1.13)
Physically active^[Contrast: physically inactive]^		0.72(0.65,0.81) ^**^		0.70(0.63,0.79) ^**^
High fat intake^[Contrast: low ]^		1.02(0.92,1.12)		1.00(0.91,1.10)
High meat intake^[Contrast: low ]^		1.15(1.05,1.27) ^**^		1.12(1.02,1.24) ^*^
High fruits & vegetables intake^[Contrast: low ]^		1.19(1.08,1.31) ^**^		1.21(1.10,1.33) ^**^
SES: Income level^[contrast: low]^	Model 3(Step1)	Model 3(Step 2)	Model 4(Step1)	Model 4(Step 2)
Medium	1.24(1.10,1.40) ^**^	1.20(1.06,1.35) ^**^	1.17(1.04,1.32) ^*^	1.15(1.01,1.29) ^*^
High	1.42(1.27,1.60) ^**^	1.35(1.20,1.51) ^**^	1.49(1.33,1.67) ^**^	1.42(1.26,1.59) ^**^
Married ^[Contrast: unmarried, divorced and widowed]^	1.40(1.16,1.69) ^**^	1.40(1.16,1.69) ^**^	1.44(1.20,1.73) ^**^	1.43(1.19,1.72) ^**^
Present smoker^[Contrast: ex-smoker& non-smoker]^		0.66(0.60,0.73) ^**^		0.77(0.70,0.86) ^*^
Present drinker^[Contrast: ex-drinker& non-drinker]^		1.06(0.95,1.17)		1.03(0.93,1.13)
Physically active^[Contrast: physically inactive]^		0.74(0.66,0.82) ^**^		0.72(0.65,0.81) ^**^
High fat intake^[Contrast: low ]^		1.03(0.93,1.13)		1.02(0.92,1.12)
High meat intake^[Contrast: low ]^		1.13(1.02,1.25) ^*^		1.09(0.99,1.20)
High fruits & vegetables intake^[Contrast: low ]^		1.22(1.11,1.34) ^**^		1.24(1.13,1.36) ^**^

^a ^All presented values are ORs with 95% confidence intervals ^b^ Defined as BMI ≥ 24 kg/m^2^^c ^Defined as WC ≥ 85 cm.

^d ^Age and age^2 ^were included in every model ^*^p < 0.05 ^**^p < 0.01.

No palpable variations were presented in adjusted odds ratios of SES indicators after the inclusion of lifestyle factors in women. Based on fitting results of final models, among two individual SES measurements, only education level was inversely associated with both types of obesity. For example, compared with individuals with primary or lower education level, the adjusted odds ratios of “general overweight and obesity” and “abdominal overweight and obesity” were 0.80 (95%CI: 0.71-0.89) and 0.85 (95%CI: 0.77-0.95) among women with secondary education level, while to women with advanced education level, the adjusted odds ratios were 0.65 (95%CI: 0.48-0.88) and 0.73 (95%CI: 0.55-0.97) instead. High fruits and vegetables intake was positively related to both types of obesity in women, too. Unlike in men, marital status was not significantly associated with obesity in women in final models (Table 4).

**Table 4:** **Logistic regression results of women, Zhejiang metabolic syndrome prevalence survey, China, 2010 **^**a**^

**Independents **^**d**^	**General overweight & obesity **^**b**^		**Abdominal overweight & obesity **^**c**^	
SES-Education level^[Contrast: primary or lower]^	Model 1(Step1)	Model 1(Step 2)	Model 2(Step1)	Model 2(Step 2)
Secondary	0.81(0.72,0.90)^**^	0.80(0.71,0.89) ^**^	0.87(0.78,0.96) ^**^	0.85(0.77,0.95) ^**^
Advanced	0.66(0.49,0.90) ^**^	0.65(0.48,0.88) ^**^	0.75(0.56,0.98) ^*^	0.73(0.55,0.97) ^*^
Married ^[Contrast: unmarried, divorced and widowed]^	1.09(0.93,1.28)	1.09(0.93,1.28)	1.14(1.11,1.16) ^**^	1.15(0.98,1.34)
Present smoker^[Contrast: ex-smoker& non-smoker]^		0.79(0.48,1.30)		0.82(0.51,1.33)
Present drinker^[Contrast: ex-drinker& non-drinker]^		1.15(0.96,1.37)		1.27(1.06,1.51) ^**^
Physically active^[Contrast: physically inactive]^		1.04(0.91,1.19)		0.90(0.79,1.03)
High fat intake^[Contrast: low ]^		0.98(0.89,1.07)		0.93(0.85,1.02)
High meat intake^[Contrast: low ]^		0.97(0.88,1.06)		0.97(0.89,1.06)
High fruits & vegetables intake^[Contrast: low ]^		1.20(1.09,1.31) ^**^		1.16(1.06,1.27) ^**^
SES-Income level^[contrast: low]^	Model 3(Step1)	Model 3(Step 2)	Model 4(Step1)	Model 4(Step 2)
Medium	0.97(0.89,1.09)	0.97(0.87,1.09)	1.01(0.90,1.13)	1.01(0.90,1.13)
High	0.99(0.89,1.10)	1.00(0.90,1.12)	1.14(1.02,1.26) ^*^	1.14(1.02,1.27) ^*^
Married ^[Contrast: unmarried, divorced and widowed]^	1.10(0.94,1.29)	1.10(0.94,1.29)	1.16(0.99,1.35)	1.16(0.99,1.36)
Present smoker^[Contrast: ex-smoker& non-smoker]^		0.77(0.47,1.27)		0.81(0.50,1.31)
Present drinker^[Contrast: ex-drinker& non-drinker]^		1.15(0.97,1.38)		1.27(1.06,1.51) ^**^
Physically active^[Contrast: physically inactive]^		1.03(0.90,1.18)		0.90(0.79,1.03)
High fat intake^[Contrast: low ]^		1.00(0.91,1.10)		0.96(0.88,1.05)
High meat intake^[Contrast: low ]^		0.96(0.87,1.05)		0.95(0.87,1.04)
High fruits & vegetables intake^[Contrast: low ]^		1.19(1.08,1.30) ^**^		1.16(1.06,1.27) ^**^

^a ^All presented values are ORs with 95% confidence intervals ^b^ Defined as BMI ≥ 24 kg/m^2^^c ^Defined as WC ≥ 80 cm.

^d^ Age and age^2 ^were included in every model ^*^p < 0.05 ^**^p < 0.01.

## Discussion

This study intended to explore association between individual SES and obesity in a Chinese adult population. Based on descriptive statistics, we found two prominent features of obesity distribution in study population. One is that, by either WGOC or WHO cut-offs, the age-and-sex standardized prevalence of general obesity in study population was considerably lower than the average level of western countries (which normally ranges from 10% to 25%), and such phenomenon has been repeatedly observed by previous Chinese studies [21-23]. The second is that, a considerable discrepancy has been found between the prevalence of general obesity and abdominal obesity in study population, say 7.6% (95%CI: 7.2%-8.0%) compared with 11.9% (95%CI: 11.4%-12.4%). We think several explanations can be made on this difference. First of all, it has been well proved that BMI can not provide information on the distribution of body fat [24], so theoretically, a certain level of discordance could exist between two indexes. Secondly, many previous studies have suggested that compared with Caucasians, Asians have a more central distribution of body fat under a given BMI [25,26], which can magnify the numerical difference between two obesity rates. Thirdly, based on literature review results, from the year 1993 to 2009, although all kinds of obesity were observed significant increases among Chinese mainlanders, the rise of abdominal obesity was the most staggering [27], such unbalanced growths can further widen prevalence gap between general obesity and abdominal obesity in Chinese populations.

In their exhaustive reviews on association between SES and obesity, Sobal [8] and Mclaren [9] concluded that, in developed societies, SES was inversely related to obesity in women, whereas in men, such association was more likely to be non-significant. In developing countries, a consistent positive correlation between SES and obesity was expected in both genders. After controlling for possible influencing factors, we found that in study population, there were significant disparities in SES-obesity association between two genders when measured by different SES indicators. In women, both general and abdominal obesity were inversely associated with education, but exhibited no significant correlation with income. In men, on the contrary, a positive association was identified only between income and obesity. Such findings indicated that the prosperity of local economy in Zhejiang province has already accelerated the transition of SES-obesity relationship from “developing country pattern” into “developed country pattern”.

It has been concluded that along with social economy development, at first the positive SES-obesity association will be attenuated, then, the negative association will gradually take a hold [9]. During the past few years, in several developing countries such as Thailand and the Philippines, a similar atypical SES-obesity relationship has also been found [11,28], which revealed that SES indicators, especially income level, were more often positively related to obesity in men, while in women, null associations were usually observed. Such results suggested that the transition of SES-obesity association in women was comparatively faster than that in men. We think Bourdieu’s “habitus theory” [29] plays a vital role in this gender-related difference. To women, a thinner figure is socially valued, and such notion can ultimately transform into formidable impetus in keeping fit figure. Some previous studies have found that, even in obesity-promoting environments, such impetus can still successfully offset the collective effect of other proponents, eventually preventing women from obesity [9]. Thus it is not surprising to find an expedited transition of SES-obesity relationship in women. But to men, a larger body size is usually valued as a sign of physical dominance and prowess, therefore social culture is comparatively tolerant to male obesity. Under such circumstance, it is much less imperative for men to pursuit thinness.

In model fitting process, after the inclusion of multiple lifestyle factors, we only discerned a minute attenuation of SES-obesity association among men, which indicated the limited and gender-specific effect of lifestyle factors as the possible intermediate variables in SES-obesity association. Such result was quite different from a previous Sweden study [30], which found a considerable proportion (18%-29%) of SES-obesity association can be explained by lifestyle factors, although the lifestyle factors in two studies were largely comparable. Besides, we also explored the independent associations between lifestyle factors and obesity. We found that in men, marriage was positively related to obesity whereas cigarette smoking was inversely related to obesity. Such results were similar to many previous studies. Moreover, among physically active male respondents, the odds of general obesity or abdominal obesity were about one quarter lower to physically inactive ones, but in women, no significant association was found between physical activity level and obesity. Such result may indicate that being physically active is a more effective way for men to prevent obesity than women. As to dietary habits, we did not find concordant significant associations between total fat intake, total meat intake and obesity. We think such result may not reflect the real situation because the daily intake amount of fat and meat among Chinese is generally low, so in our study, the quantity difference between dichotomized “high intake” and “low intake” subgroups may be too small to detect the real effect. However, to fruits and vegetables, we did observe an expected positive relationship between intake amount and obesity in both genders. We think the main reason that fruits and vegetables intake can be a plausible surrogate for total energy intake among Chinese is that China is an agricultural country, the prices of fruits and vegetables are comparatively low, so they are generously consumed in every meal with staples (commonly rice or wheaten food), in this instance, fruits and vegetables intake amount can be positively related to staples intake amount. And in China, staples are the predominant sources of daily energy intake.

Several limitations of this study should be noticed. Firstly, it was a cross-sectional study, which means we can not draw any causal-effect conclusion based on study results. Secondly, although the response rate was high (91.2%), it turned out that some traits of participants were still slightly different from general population of Zhejiang, such trivial discrepancies could still bring bias to study results. Thirdly, despite fruits and vegetables intake can be an ideal surrogate for daily energy intake, it is very likely that the results may exhibit a certain degree of discordance if we were able to use daily energy intake in the first place. At last, considering the huge discrepancies in demographic characteristics, lifestyle pattern and economic strength between different regions of China, our study results might not be applicable to other Chinese subpopulations as well as the whole Chinese population.

Despite all limitations, the major findings of our study have important secular significance. China is a developing country with highly unbalanced regional development. In economically prosperous areas, like Zhejiang, based on our findings, SES-obesity association has already resembled to which in developed societies. But to those impoverished inland provinces, such relationship can be totally different. Thus it is of great importance to implement SES-obesity association studies in other parts of China, in order to accurately target high risk groups of obesity, and successfully implement intervention measures. Furthermore, many previous studies have revealed that macro environment (neighborhood and area) was closely related to the well-being of residents [31,32], and some scholars have already explored its relationship with obesity in specific subpopulations (mostly in children) [33,34], but nearly all of these studies were implemented in developed countries, so it is also of great necessity to carry out relevant studies in Chinese populations in the near future.

## Conclusions

In this study, we found that in women, a significant inverse association was existed between education and obesity, while in men, income rather than education was positively related to obesity. This atypical pattern in study population reflected the on-going transition of SES-obesity association in economically prosperous areas of China, which was propelled by social economy transformation. Based on study results, high-income men and poorly-educated women were high risk groups of obesity in Zhejiang province, which need intense focuses. More studies addressing SES-obesity relationship in different Chinese subpopulations are desperately needed.

## Competing interests

The authors declare no competing interests.

## Authors’ contributions

ZY conceptualized the whole study, YX performed data analysis and drafted the manuscript, NZ gave instructions on data analysis, HW, JZ,QH, DS, MZ, LW, XZ, RH, MY helped with data collecting and sorting. All authors read and approved the final manuscript.

## Pre-publication history

The pre-publication history for this paper can be accessed here:

http://www.biomedcentral.com/1471-2458/13/355/prepub
